# Investigation of the Bystander Effect on Cell Viability After Application of Combined Electroporation-Based Methods

**DOI:** 10.3390/ijms26052297

**Published:** 2025-03-05

**Authors:** Neringa Barauskaitė-Šarkinienė, Vitalij Novickij, Saulius Šatkauskas, Paulius Ruzgys

**Affiliations:** 1 Research Institute of Natural Sciences and Technology, Vytautas Magnus University, Universiteto Str. 10, LT-53361 Kaunas, Lithuania; neringa.barauskaite-sarkiniene@vdu.lt (N.B.-Š.); saulius.satkauskas@vdu.lt (S.Š.); 2 Institute of High Magnetic Fields, Vilnius Gediminas Technical University, LT-03227 Vilnius, Lithuania; vitalij.novickij@imcentras.lt; 3 Department of Immunology, State Research Institute Centre for Innovative Medicine, Santariškių 5, LT-08410 Vilnius, Lithuania

**Keywords:** electroporation, bleomycin electrotransfer, bystander effect, calcium electroporation

## Abstract

Electrochemotherapy (ECT) uses electroporation to enhance drug delivery into tumor cells, triggering bystander effects like immunogenicity and cell death. This study investigated bystander effects in vitro in 4T1 breast cancer cells following various electroporation treatments: reversible (1400 V/cm, 100 µs) bleomycin electrotransfer, irreversible (2800 V/cm, 100 µs) bleomycin electrotransfer, and calcium electroporation, including combinations. Conditioned media from treated cells (12–72 h incubation) were transferred to untreated cells, and viability was assessed via metabolic activity, cell count, and colony formation. A scratch assay evaluated wound healing. The bystander effect dramatically reduced colony formation, reaching 0% after bleomycin and calcium electrotransfer, and 2.37 ± 0.74% after irreversible electroporation (IRE). Metabolic activity decreased to 18.05 ± 6.77% and 11.62 ± 3.57% after bleomycin and calcium electrotransfer, respectively, and 56.21 ± 0.74% after IRE. Similarly, cell viability measured by flow cytometry was 10.00 ± 1.44%, 3.67 ± 0.32%, and 24.96 ± 1.37% after bleomycin electrotransfer, calcium electrotransfer, and IRE, respectively. Combined analysis of these effects yielded comparable results. Conditioned media, particularly from bleomycin electrotransfer and calcium electroporation, significantly reduced cell number, metabolic activity, and colony formation, demonstrating a strong bystander effect. Wound healing was also significantly delayed in groups exposed to conditioned media.

## 1. Introduction

Conventional cancer treatment methods, such as surgery, chemotherapy, and radiotherapy, while effective, lack the precision to selectively target cancer cells, resulting in the unintended disruption of healthy cells and undesirable side effects for patients [1]. Consequently, there is a prominent emphasis in the field of scientific research on advancing therapeutic technologies and enhancing the efficacy of cancer treatment. This endeavor has yielded innovative anticancer therapies that hold significant potential for achieving complete tumor regression. One such strategy involves the precise delivery of molecules, such as anticancer drugs, directly into tumor cells through the application of pulsed electrical fields (PEFs) [2,3,4]. This cancer treatment method is termed electrochemotherapy (ECT) and is widely employed in clinical settings [5]. ECT operates on the principle of electroporation, wherein the cellular membrane transiently becomes permeable to hydrophilic anticancer drugs such as bleomycin (BLM), when subjected to electric fields [6]. ECT with BLM proves to be very efficient [7]. This method was clinically tested and used for quite a while already [8]. Nevertheless, an alternative to bleomycin electrotransfer emerged, involving the use of Ca^2+^ ions, which serve as universal signaling mediators regulating numerous cellular functions, including exocytosis, metabolism, gene expression, and the cell cycle [9,10]. It is known that high concentrations of Ca^2+^ ions exhibit cytotoxic effects on cells [11]. Thus, by electroporating cells in a medium supplemented with Ca^2+^ ions, it becomes possible to induce cell death. Additionally, Ca^2+^ electroporation induces secondary effects, such as an enhanced immunogenic response in cancer cells [12].

While Ca^2^⁺ ions can be beneficial in combination with electroporation, their effects are not always positive. Our previous work demonstrated the importance of calcium in membrane repair, showing significant enhancement at concentrations above 0.1 mM [13]. We also established that cells effectively remove excess calcium after electroporation when extracellular Ca^2^⁺ is below 1 mM. However, this ability is lost at higher extracellular concentrations (above 1 mM), leading to irreversible calcium dysregulation and in vitro cell death.

Another type of cancer treatment via application of electric fields is referred as irreversible electroporation (IRE). High intensity electric fields can trigger cell death via IRE when the damage to the cell plasma membrane is so severe that the cell cannot recover [14]. This is a key mechanism for IRE to occur. Presently, IRE has shown an extremely promising results in anticancer treatment clinical trials against liver [15], prostate [16], pancreatic [17], gastric [18], and other cancers.

To date, limited research has successfully explored the potential transformation of these localized methods into systemic treatments [19]. Nevertheless, the idea for electroporation anticancer treatments to outgrow local treatment status has already been present for a while [20]. The prospect of such advancement in electroporation-based anticancer therapies stems from the directly affected cell response to the electroporation-based treatments. The affected cells secrete various signal molecules in the vicinity surrounding them, hence triggering additional effects like immunogenicity to the treated tumor or the killing effect to the neighboring cells [21]. This phenomenon is known as the “bystander” effect [22]. There are very few articles published on the bystander effect in the field of electroporation [19,21,23]. Nevertheless, the resemblance to radiotherapy, a conventional local cancer treatment involving the direct application of ionizing radiation to the tumor site could broaden the knowledge on the bystander effect mechanism. It has been observed that cells release signaling molecules into their vicinity after exposure to ionizing radiation, and these molecules adversely affect neighboring cells not directly exposed to radiation [24].

The little knowledge on the bystander effect after electroporation is the following. During the in vitro electroporation-based experiments, it was observed that the bystander effect occurs in CHO-K1 noncancer cell line following both irreversible electroporation (resulting in a positive viability effect) and the electrotransfer of the anticancer drug bleomycin (resulting in a negative viability effect) [21]. Furthermore, it is already shown that the bystander effect may potentially augment the abscopal effect in vivo [19].

Despite growing knowledge surrounding the bystander effect, it remains unclear whether this phenomenon can be harnessed by combining various electroporation-based methods, including irreversible electroporation, calcium electroporation, and bleomycin electrotransfer. Furthermore, the impact of the bystander effect combined effect of cell migration and proliferation requires further investigation.

In this article, we present novel findings regarding the bystander effect and the response of the 4T1 cell line to treatments involving irreversible electroporation, bleomycin electrotransfer, and calcium electroporation. Additionally, we have identified additional effects arising from various treatment combinations. Also, the possible impact of the bystander effect on wound closure is shown by using scratch test in vitro.

## 2. Results

**Figure 1:**
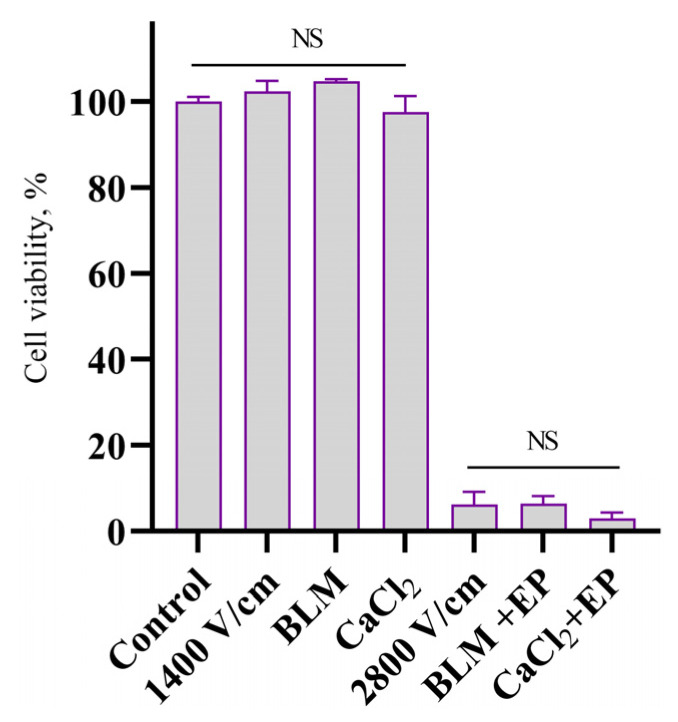
4T1 cell viability after 1400 V/cm, 1 HV, BLM alone, and CaCl_2_ alone and treatment with bleomycin (20 nM) electrotransfer (1400 V/cm, 1 HV), CaCl_2_ (1mM) electroporation (1400 V/cm, 1 HV), and irreversible electroporation (IRE) (2800 V/cm, 1 HV). The graph shows cell viability after 6 days of incubation, % of control cell colony number. The graph shows the averages (n = 6) ± standard error of mean (SEM). Statistical significance value here: NS—not significant.

First of all, the impact of CaEP, BLM electrotransfer, and IRE on 4T1 cell viability after standard electroporation had to be assessed. The cell viability was measured with clonogenic assay. Results presented in Figure 1 indicate that bleomycin electrotransfer eradicates most of the cells with only 6.4 ± 1.85% of survival rate. The same trend is seen in experiments with 1 mM CaCl_2_ and irreversible electroporation, correspondingly, having 3.0 ± 1.4% and 6.2 ± 3.0% remanent cells. The effects of electroporation alone, bleomycin alone, and CaCl_2_ alone on cell viability were also evaluated, and no significant effects were observed.

**Figure 2:**
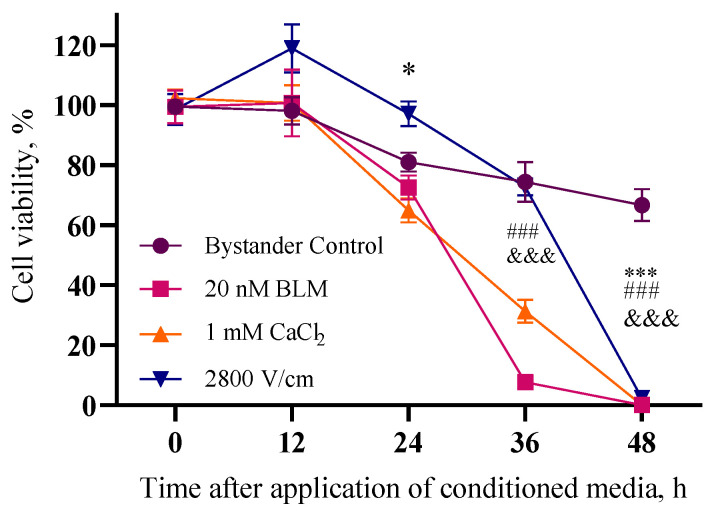
The influence of the bystander effect for 4T1 cell viability after 0–48 h in the bystander control group (no treatment with electrical pulses), after 20 nM BLM electrotransfer (1400 V/cm, 1 HV), irreversible electroporation (2800 V/cm, 1 HV), and calcium electroporation (1 mM) (1400 V/cm, 1 HV). The graph shows cell viability after 6 days of incubation, % of control cell colony number. The graph shows the averages (n = 6) ± standard error of mean (SEM). Statistical significance values are indicated as follows: *, *p* < 0.05; ***, ###, or &&&, *p* < 0.001.

Having done this, the next matter that we needed to address was incubation time for making conditioned medium. Inappropriate timing there might cause undesired viability changes in control cells. Therefore, the next experiment was conducted in order to evaluate conditioned media incubation timing in a range from 0 to 48 h. Results are presented in Figure 2. Cells in the bystander control experimental group were only incubated in conditioned media where cells were not affected with electroporation. As seen in Figure 2, we obtained a bystander control cell viability decrease during the incubation time and after 48 h viability of the cells remained 66.7 ± 5.2%. In experiments with BLM and CaCl_2_ electrotransfer, we could obtain similar results. The bystander effect does not occur after 24 h of incubation—viability of cells is 72.5 ± 4.0% with BLM and 64.9 ± 4.0% with CaCl_2_. After 48 h, cell viability in both cases is minimal (0%), so, in this case we have a negative bystander effect. We could obtain slightly different results with irreversible electroporation—after 12 h, viability of cells increased (118.9 ± 8.0%), but then started to decrease (97.1 ± 4.2% after 24 h) and after 48 h, viability of the cells was minimal (2.4 ± 0.9%). This intriguing result of cell viability increase was unexpected since the measurements were performed by using a clonogenic assay.

**Figure 3:**
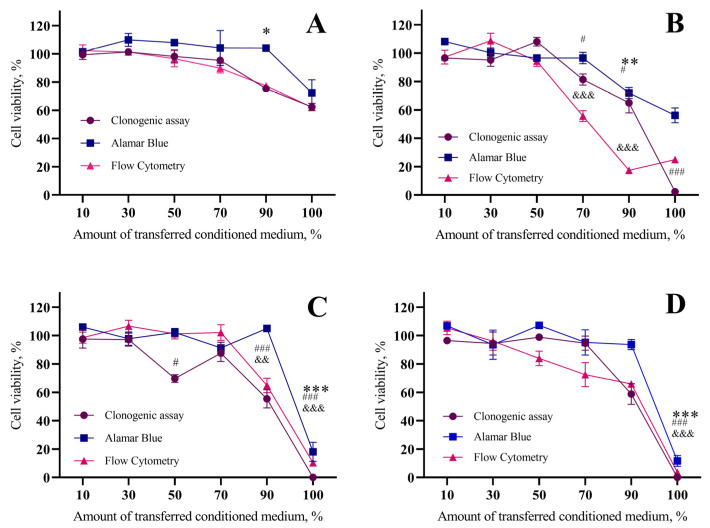
The influence of the bystander effect for 4T1 cell viability and dependence on the amount of transferred bystander medium (transferred after 48 h) in the bystander control group (no treatment with electrical pulses) (**A**); after irreversible electroporation (2800 V/cm, 1 HV) (**B**); 20 nM BLM electrotransfer (1400 V/cm, 1 HV) (**C**) and calcium electroporation (1mM) (1400 V/cm, 1 HV) (**D**). The graph shows the dependence of cell viability on the amount of transferred bystander medium, after 6 days of incubation (clonogenic assay) and 48 h of incubation (Alamar Blue and flow cytometry), % of control group value. The graph shows the averages ± standard error of mean (SEM). Statistical significance values are indicated as follows: * or #, *p* < 0.05; ** or &&, *p* < 0.01, ***, ###, or &&&, *p* < 0.001.

In the subsequent experiments, we assessed the dependence of the bystander effect on the amount of transferred affected media after a 48 h period. In this study, we employed three distinct methods for measuring cell viability: clonogenic assay, flow cytometry, and Alamar Blue (for assessing metabolic activity). The results are illustrated in Figure 3.

Figure 3A displays the results of the bystander control experiment. We observed similar trends across all employed cell viability measurement methods. Specifically, when 50% of the transferred media was used, cell viability remained at 98.2 ± 4.6% (clonogenic assay), 107.9 ± 1.0% (Alamar Blue), and 96.6 ± 5.7% (flow cytometry). However, when 100% of the transferred media was utilized, cell viability decreased, maintaining values of 62.4 ± 2.6% (clonogenic assay), 72.4 ± 9.4% (Alamar Blue), and 62.1 ± 0.8% (flow cytometry).

In Figure 3B, we present the results of bystander experiments following treatment with irreversible electroporation. When 50% of the transferred media was used, cell viability remained at 108.09 ± 3.1% (clonogenic assay), 96.5 ± 1.2% (Alamar Blue), and 94.4 ± 3.7% (flow cytometry). However, when 100% of the transferred media was employed, cell viability decreased significantly and remained at 2.4 ± 0.7% (clonogenic assay), 56.2 ± 5.2% (Alamar Blue), and 25.0 ± 1.4% (flow cytometry).

Figure 3C displays the results of bystander experiments conducted after treatment with 20 nM BLM. Under conditions where 50% of the transferred media was used, cell viability was maintained at 69.7 ± 2.8% (clonogenic assay), 102.2 ± 2.8% (Alamar Blue), and 101.3 ± 3.6% (flow cytometry). However, when 100% of the transferred media was applied, cell viability decreased drastically, reaching 0% (clonogenic assay), 18.1 ± 6.8% (Alamar Blue), and 10.4 ± 1.4% (flow cytometry).

Figure 3D presents the outcomes of bystander experiments following treatment with 1 mM CaCl_2_. When 50% of the transferred media was utilized, cell viability was sustained at 98.9 ± 0.2% (clonogenic assay), 107.2 ± 1.4% (Alamar Blue), and 83.9 ± 5.0% (flow cytometry). Conversely, when 100% of the transferred media was employed, cell viability plummeted to 0% (clonogenic assay), 11.6 ± 3.8% (Alamar Blue), and 3.7 ± 0.3% (flow cytometry).

**Figure 4:**
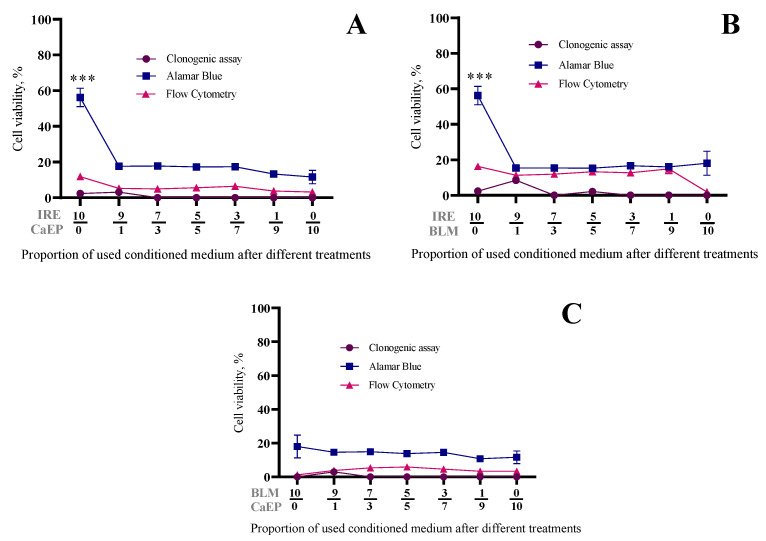
The influence of bystander effect for 4T1 cell viability. Dependence on combination of different treatments (CaEP + IRE (**A**); BLM + IRE (**B**); CaEP + BLM (**C**)) and amount of transferred conditioned medium (transferred after 48 h). The graph shows dependence of cell viability on the amount of transferred conditioned medium, after 6 days of incubation (clonogenic assay) and 48 h of incubation (Alamar Blue and flow cytometry), % of control group value. The graph shows the averages ± standard error of mean (SEM). Statistical significance values are indicated as follows: *** *p* < 0.001.

Lastly, we assessed the dependence of cell viability on a combination of different treatments and the amount of transferred bystander medium (transferred after 48 h). The results are presented in Figure 4. Additionally, throughout these experiments, we employed three distinct methods for measuring cell viability: clonogenic assay, flow cytometry, and Alamar Blue (for assessing metabolic activity).

In Figure 4A, we present the experimental results following combinations of bystander media with CaCl_2_ and IRE treatments. Notably, consistent trends were observed across all utilized cell viability measurement methods. When 50% of the transferred media was used, cell viability remained at 0% (clonogenic assay), 17.3 ± 0.2% (Alamar Blue), and 5.6 ± 0.3% (flow cytometry). Conversely, when 100% of the transferred media was employed after treatment with calcium, cell viability decreased and remained at 0% (clonogenic assay), 11.6 ± 3.8% (Alamar Blue), and 3.3 ± 0.2% (flow cytometry). Similar trends were also observed with combinations of BLM and IRE (Figure 4B) and BLM and CaCl_2_ (Figure 4C).

**Figure 5:**
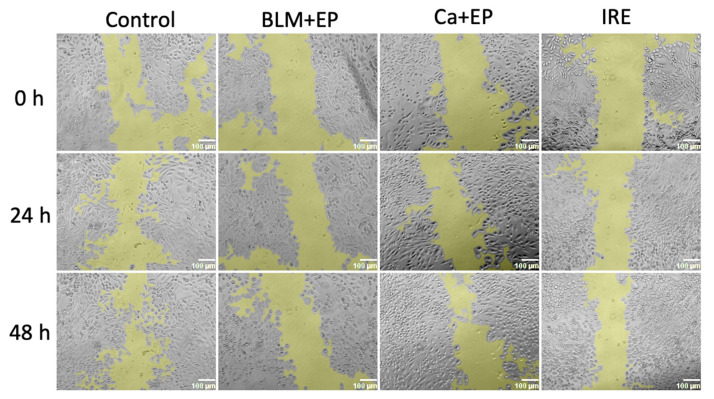
The influence of the bystander effect on 4T1 cell wound healing after CaEP, BLM electrotransfer, and irreversible electroporation. The images illustrate the ability of 4T1 cells to close wounds under the influence of the bystander effect following calcium electroporation (CaEP), bleomycin (BLM) electrotransfer, and irreversible electroporation. The images were taken using an inverted light microscope (Kern OCO-255) at intervals of 0, 24, and 48 h after the affected medium was transferred. The area of the surface not covered by cells (the wound) is highlighted in yellow.

**Figure 6:**
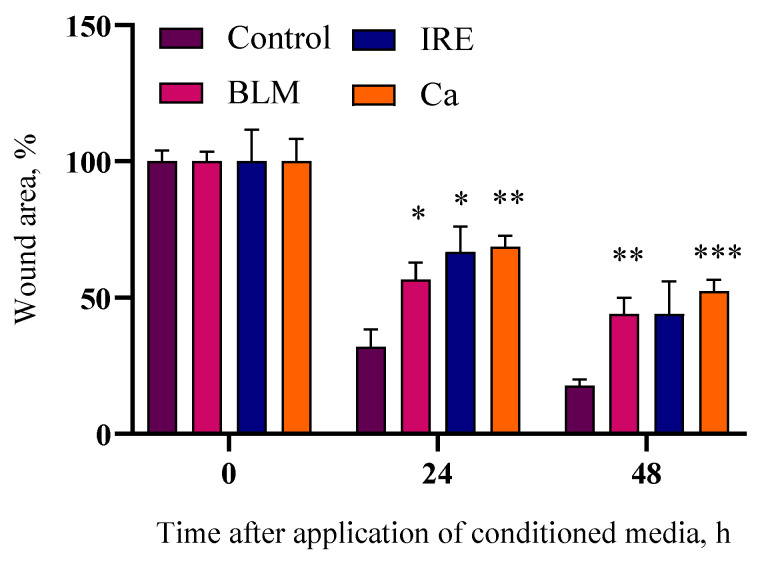
The influence of the bystander effect on 4T1 cell wound healing after CaEP, BLM electrotransfer, and irreversible electroporation. This figure illustrates the percentage of wound closure at 0, 24, and 48 h after transferring the affected medium, compared to the initial wound size at time 0 h. The wound closure percentage was calculated using ImageJ (2.14.0/1.54f) software with the Wound Healing Size Tool plugin. The graph presents the averages ± standard error of the mean (SEM). Statistical significance values are indicated as follows: * *p* < 0.05, ** *p* < 0.01, *** *p* < 0.001, NS—not significant.

During anticancer therapies based on electric fields using needle-type electrodes, a large part of the affected tissue is not directly impacted by the electric field. However, it is not known whether the bystander effect influences this tissue. Therefore, further research aimed to evaluate whether the bystander effect affects the wound healing process. Images reflecting the obtained results are shown in Figure 5, and the reduction in the surface area not covered by cells (wound area) as a percentage, compared to the initial wound area, is shown in Figure 6.

While ImageJ (2.14.0/1.54f) software provides a reasonably accurate assessment of wound area, it cannot capture all relevant differences. Cell morphology and distribution, for example, shows differences that enables further analysis of the images. Comparing the IRE and control groups 48 h post-treatment reveals a key distinction: the control group exhibits numerous small, unattached cells within the wound center, a feature absent in the IRE group. A similar, albeit less pronounced, phenomenon is observed in the BLM + EP group, where isolated cells are present, but confined to the wound periphery. The calcium EP group, however, appears similar to the control group regarding the presence of small cell clusters in the central wound area.

The results after counting the area can be interpreted differently. It can be seen in Figure 6 that the cells in the control group covered 68.1 ± 6.3% of the wound area after 24 h and 82.2 ± 2.1% after 48 h. The cells exposed to the bystander medium after treatment with electric fields and the anticancer drug bleomycin covered 43.3 ± 6.2% of the wound area after 24 h and 55.9 ± 5.9% after 48 h. The greatest impact on the wound healing process was observed in the bystander medium after irreversible electroporation (IRE) and calcium electroporation (CaEP), where only 33.2 ± 9.3% (IRE) and 31.3 ± 4.1% (CaEP) of the wound area were covered after 24 h, and 55.9 ± 11.9% (IRE) and 47.6 ± 4.2% (CaEP) after 48 h. These results suggest that the bystander effect generated after exposure to electric fields influences the wound healing process and adversely affects not only cell proliferation but also migration, which is crucial for tissue formation.

## 3. Discussion

To investigate this, we first conducted experiments to confirm the effectiveness of our electroporation protocols. All utilized methods reduced cell viability to less than 10%, prompting further investigation with a clonogenic assay. This assay reveals the loss of colony-forming ability, indicating cell death or irreversible damage preventing further division. These initial experiments were crucial as only three previous studies mentioned electroporation triggered bystander effect, and none broadly investigated them in 4T1 cells.

Subsequently, we focused on developing protocols to demonstrate the bystander effect. This proved challenging due to variations in cell sensitivity, doubling time, size, and other characteristics. We hypothesized that dying cells release signals into the surrounding media, potentially inducing bystander effects. To test this, we cultured unaffected cells in conditioned media harvested from cultures of directly electroporated cells and performed clonogenic assays.

Figure 2 demonstrates a significant viability change in unaffected cells exposed to conditioned media (“bystander control”) compared to control grown in standard growth media. While the precise mechanism remains unclear, we suspect nutrient depletion in the conditioned media might play a role, as a high density of cells were cultured in it for an extended period.

Therefore, the incubation time of directly affected cells in the conditioned media is crucial. Our results showed that bystander effects become apparent after 36 to 48 h of conditioning of 4T1 cells. We chose a 48 h conditioning period for subsequent experiments due to the more pronounced effect and the lack of significant viability reduction in the bystander control.

Interestingly, clonogenic assay results revealed a notable, albeit not statistically significant, trend of increased viability after 12 to 24 h of exposure to conditioned media made with cells after irreversible electroporation application. This intriguing observation, repeated across 12 independent experiments with three replicates each, consistently showed increased cell viability after irreversible electroporation treatment. This suggests a genuine biological phenomenon rather than experimental error.

We hypothesize that this might be attributed to a stress-induced cellular response. Previous research has demonstrated that stress can enhance cell metabolic activity and migration [20]. We speculate that a similar phenomenon might be at play here, potentially enhancing survival and migration. This observation raises concerns about the possibility of a pro-metastatic effect triggered by the bystander effect, specifically after irreversible electroporation. Notably, this phenomenon was not observed after bleomycin electrotransfer or calcium electroporation.

In our investigation, we opted for a 48 h conditioning period to generate conditioned media for bystander effect experiments. Previous work with CHO cell lines indicated that bystander effect-induced viability changes might be contingent upon the dilution of conditioned media from dying cells [20]. This observation stemmed from experiments where diluting the conditioned media with standard growth media led to a significant increase in viability of the indirectly affected cells, even at slight dilutions. This raised the possibility that nutrient depletion in the conditioned media contributed to the observed viability changes.

To further explore this phenomenon, we conducted clonogenic assays alongside metabolic activity measurements (Alamar Blue assay) and cell counts at 48 h post-treatment (flow cytometry). This multifaceted approach allowed us to assess the bystander effect’s impact on cell division, metabolic activity, and colony formation potential. Our findings revealed largely consistent effects across all three assays, with some minor variations.

Metabolic activity emerged as the least affected parameter. However, this assay presented unique challenges. Initially, measuring conditioned media alone yielded a positive result. To mitigate this, we replaced the conditioned media with phosphate buffer saline (PBS) immediately before Alamar Blue dye incubation (2 h before measurement). Although this adjustment addressed the artifact, it likely resulted in an underestimation of the true positive effect. Despite this limitation, the overall trend observed in the metabolic activity assay remained consistent with the other assays.

As depicted in Figure 3, cell viability increased with diluting conditioned bystander media. As previously noted, the control group exposed to conditioned media from unaffected cells exhibited a slight decrease in viability compared to other groups. Interestingly, the metabolic activity of cells exposed to conditioned media from irreversible electroporation differed from that observed with bleomycin electrotransfer and calcium electroporation. The metabolic activity profile after irreversible electroporation more closely resembled the control group. This observation aligns with our previous finding that irreversible electroporation unexpectedly induced cell migration (Figure 2). It is plausible that the observed effects on metabolic activity are also influenced by this migration phenomenon.

Furthermore, the 48 h time point might be insufficient for the effects of conditioned media from dying cells, following irreversible electroporation, to be fully observed in indirectly affected cells. This notion is supported by the clonogenic assay results, which showed no colony formation after 6 days and a low cell count at 48 h post-incubation with conditioned media from irreversible electroporation. Notably, the decline in cell number was far more pronounced than the reduction in metabolic activity. This suggests that surviving cells exhibited drastically elevated metabolic activity, potentially indicative of a stress response. This phenomenon was not observed with bleomycin electrotransfer or calcium electroporation when cells were cultured in 100% conditioned media. However, the same increase in metabolic activity in surviving cells was apparent upon the addition of 10% standard growth media.

Intriguingly, diluting the conditioned media from irreversible electroporation by 10% yielded another unexpected result: despite a substantial reduction in cell number (around 20%), the clonogenic assay indicated approximately 70% viability. This observation might suggest the induction of cellular senescence, a phenomenon we previously observed when analyzing colony sizes after exposure to conditioned media from irreversible electroporation and bleomycin electrotransfer. Notably, those experiments revealed significantly smaller colonies compared to the control group.

Our findings from media dilution experiments underscore the importance of the dilution of conditioned media from directly affected cells in mediating the bystander effect. In a tissue context, this dilution gradient can be considered analogous to the distance between directly and indirectly affected cells. This hypothesis is further bolstered by the fact that electroporation exerts its effects locally, implying that the bystander effect will be most pronounced in close proximity to directly affected cells.

In light of the growing trend toward combining different electroporation-based therapies, such as irreversible electroporation with calcium electroporation or bleomycin electrotransfer, understanding the bystander effects of these combinations becomes crucial. To date, only one study has briefly touched upon the bystander effect in the context of combined calcium electroporation and bleomycin electrotransfer [18]. However, this work focused primarily on abscopal effects rather than a dedicated analysis of the bystander effect. Furthermore, irreversible electroporation was not included, and dilutions were limited to 50% conditioned media from cells treated with either bleomycin electrotransfer or calcium electroporation.

Here, we conducted a comprehensive analysis involving various dilutions and incorporated not only clonogenic assays but also metabolic activity measurements and flow cytometry-based cell counts. Our findings, presented in Figure 4, demonstrate that combining conditioned media from different treatments enhances the bystander effect compared to unconditioned media. This conclusion is evident when comparing Figure 3 and Figure 4. Specifically, Figure 4A illustrates that all ratios of conditioned media from bleomycin electrotransfer and calcium electroporation similarly affect cell number, metabolic activity, and colony formation, leading to a decrease in all three parameters.

As previously discussed, the metabolic activity following irreversible electroporation was consistently higher than both clonogenic assay and flow cytometry results. However, the presence of at least 10% conditioned media from either bleomycin electrotransfer or calcium electroporation consistently reduced metabolic activity to levels comparable to those observed in clonogenic and flow cytometry assays. This suggests that the bystander effect triggered by irreversible electroporation, in conjunction with either bleomycin electrotransfer or calcium electroporation, exerts a more pronounced negative effect on cellular metabolic activity. This finding, reported here for the first time, warrants further investigation.

Electroporation-triggered bystander effects remain poorly understood. The existing literature suggests that these effects manifest after successful electroporation treatment, hypothesizing that the mechanism relies on secreted signaling molecules from dying, electroporated cells [21,23]. Given the limited publications on bystander effects after electroporation, insights from another local physical method, ionizing radiation, can be informative. The bystander effect mechanism in ionizing radiation is associated with signal transduction via cytokines, reaching recipient or bystander cells through the gap junctions, direct cytokine secretion into the extracellular medium, and via extracellular vesicles (EVs) [25]. Candidate signaling molecules generated by ionizing radiation include TGF-β [26,27], NFκB, MAPK, and COX-2 [28]. Furthermore, recent evidence indicates that EVs can transfer gasdermin-based pores from directly affected cells to bystander cells, triggering pyroptosis [29].

The bystander effect after electroporation may also involve exosomes or direct molecular exocytosis. Electroporation has been shown to substantially increase the secretion of EVs up to 20 h post-treatment [30]. This study also shows that electroporation can alter the microRNA composition within these exosomes.

The role of the bystander effect in cell migration is complex and potentially contradictory. The contents of EVs can contribute to tissue healing or even tumor metastasis [30], but can also decrease cell viability by transferring inflammatory mediators [29]. This duality may explain the observed positive and negative effects of the bystander effect [25].

The wound healing assay, or scratch test, provides a valuable tool to assess the combined effects of cell migration and proliferation on wound closure within a cell monolayer [31]. While typically employed to evaluate the impact of treatments like drugs or growth factors, we utilized this assay to investigate the influence of the bystander effect on wound closure.

Our findings revealed a significant delay in wound closure in groups exposed to conditioned media from cells subjected to irreversible electroporation, bleomycin electrotransfer, or calcium electroporation, compared to the control group. This suggests that the bystander effect demonstrably hinders the collective ability of cells to close a wound. Furthermore, wound healing images reveal variations in cell distribution. The control group, and to some extent the CaEP group, display distinct cell islets within the wound bed, potentially indicating cell migration like in the control. Conversely, the absence of these islets in other groups suggests impaired migration. However, the presence of these islets does not fully correlate with wound closure, as evidenced by a comparison of Figure 5 and Figure 6.

While this observation might suggest a potential impact on in vivo tissue wound healing surrounding needle electrodes used for electric field application, it is crucial to acknowledge the inherent limitations of the scratch assay. This simplified model lacks the complex interplay of various cell types and intricate extracellular matrix components present in a real wound.

Nevertheless, the wound healing assay provides valuable insights into the collective behavior of cell populations. Our control group exhibited approximately 70% wound closure after 24 h, reaching roughly 80% by 48 h. The observed significant delays in wound closure in groups exposed to bystander-conditioned media underscore the potential impact of this phenomenon on the wound healing process, warranting further investigation in more complex models.

## 4. Materials and Methods

### 4.1. Cultivation of the Cells

Experiments were performed using breast cancer cell line 4T1. Cells were cultured in RPMI media (Sigma-Aldrich, Saint Louis, MO, USA), supplemented with 1% penicillin-streptomycin (Sigma-Aldrich) and 10% fetal bovine serum (Sigma-Aldrich). Every two to three days and the day before an experiment, the cells were passaged (having less than 80% of confluency). Phosphate buffer saline (PBS) was used to wash all the remains of old media, then cells were incubated in 37 °C for 2 min with trypsin–EDTA solution. After this process, cells were centrifuged for 2 min at 1000 rpm (Biosan, Riga, Latvia, LMC-3000). Following centrifugation, cells were resuspended in 1 mL of growth media. Then, cell concentration was evaluated, and appropriate number of cells were put in 96 mm tissue culture plates (TPP) with 10 mL of growth medium incubated at 37 °C in a humidified incubator with 5% CO_2_.

### 4.2. Electroporation

The pulse generator (Amber Charge, Kaunas, Lithuania) was used to generate 280 or 560 V square wave electric pulses. Stainless steel plate electrodes with a 2 mm gap were used as an applicator and the load. For reversible electroporation, 1 square electric pulse with an intensity of 1400 V/cm and duration of 100 μs was used and for irreversible electroporation, 1 square wave pulse with an intensity of 2800 V/cm and duration of 100 μs was employed in the study.

Before the experiments, cells were detached from the surface of the plate using trypsinization. After that, cells were centrifuged at 1000 rpm (Biosan, LMC-3000) and resuspended in 1 mL of laboratory-made low conductivity electroporation media (0.1 S/m, 270 mOsm, pH = 7; 242.19 mM sucrose, 5.59 mM Na_2_HPO_4_, 1.73 mM MgCl_2_, 3.00 mM NaH_2_PO_4_) for bleomycin electrotransfer and irreversible electroporation. For calcium electroporation, HEPES medium was used. This medium is composed of 10 mM HEPES (Lonza, Basel, Switzerland), 250 mM sucrose (Sigma-Aldrich), and 1 mM MgCl_2_ (Sigma-Aldrich, Saint Louis, MO, USA) pH 7.1, 0.01 S/m.

An amount of 45 μL of cell suspension (2 million cells/mL) was supplemented with 5 μL bleomycin (200 nM) or calcium (10 mM) suspension. The mixture of this medium was placed in between electrodes and pulsed with electric fields. Then, this suspension was placed in a 6-well plate (TPP, Schaffhausen, Switzerland), incubated for 10 min, and 2 mL of growth media was put on the electroporated cells.

### 4.3. Conditioned Media Generation

The EP-affected cells were plated into a 6-well plate (TPP, Switzerland) with 5 experimental points (0.45 million cells) into each of the wells and incubated for 10 min. Afterwards, an amount of 1000 μL of growth media (200 μL for each experimental point) was poured on the cells and the plate was incubated for the appropriate time (12–72 h). If the incubation time was not the variable, then incubation was conducted for 48 h at a temperature of 37 °C.

After the incubation period, the growth media from directly affected cells were collected and centrifuged twice at 1000 RPM, 2 min for one centrifugation (Biosan LMC-3000, R-12/15). The absence of cells in the conditioned media was additionally verified by adding 35 μL of the media to a hemocytometer and inspecting it visually under the microscope. Such a conditioned medium was used to cause the bystander effect on directly untreated cells. For the colony formation assay, 2000 μL of conditioned media (from 10 experimental points) was used per plate, which contained 400 cells. For Alamar Blue and flow cytometry experiments, 400 μL of conditioned media (from 2 experimental points) was used per well of a 24-well plate.

### 4.4. Measurement of Bystander Effect

After incubation of cells directly unaffected with EP with conditioned media, we used three different methods to measure the bystander effect—cell colony formation test; cell metabolic activity with Alamar Blue; and measuring the amount of the remaining cells with flow cytometry.

#### 4.4.1. Cell Colony Formation Test

For the measurement of the bystander effect influence for untreated cells and their ability to form colonies, we used 400 cells and a 40 mm Petri dish (TPP) supplemented with 2 mL RPMI growth medium. Cells were plated 24 h before the treatment with conditioned media. After 24 h, conditioned media were transferred to untreated cells. Then, the cells were incubated at 37 °C with the conditioned media for 5 days. Afterwards, crystal violet solution (40% EtOH, 20% dH_2_O, 40% crystal violet dye (Sigma-Aldrich)) was used to stain the colonies. Afterwards, the petri dishes with colonies were imaged. The counting of stained colonies was performed with ImageJ (2.14.0/1.54f) software (National Institute of Health, Bethesda, MD, USA); the number of the colonies was assessed in accordance with instructions supplied by the software’s developers. Then, the number of colonies was normalized with the control and presented in graphs.

#### 4.4.2. Alamar Blue Cell Viability Assay

The metabolic activity of cells could be quantitatively measured with Alamar Blue dye, which contains resazurin, a REDOX (oxidation-reduction) indicator. The oxidized form of this indicator is non-fluorescent (blue color), and, in case of cell enzymatic activity, this chemical reduction takes place and the creation of resorufin is observed. This can be measured as the resorufin is highly fluorescent, hence the metabolic activity of cells could be evaluated.

After incubation of directly affected cells, the growth medium was collected and centrifuged twice. Then, 2500 untreated cells were plated into each well on a 24-well plate and conditioned medium (400 μL for each well) was poured onto unaffected cells. Cells were incubated at 37 °C for 48 h. After that time, the medium was changed with 360 µL of the phosphate buffer saline (PBS) and 40 μL of Alamar Blue reagent was added into each well and the plate was incubated for 2 h (at 37 °C). After incubation, fluorescence (525 nm excitation, 590 nm emission) was measured with a Tecan GENios Pro Microplate Reader (Männedorf, Switzerland). For calibration and control group, only growth media (without cells) and cells with unaffected growth medium were used and results were compared.

#### 4.4.3. Quantity Measurement of Cells with Flow Cytometry

**Figure 7:**
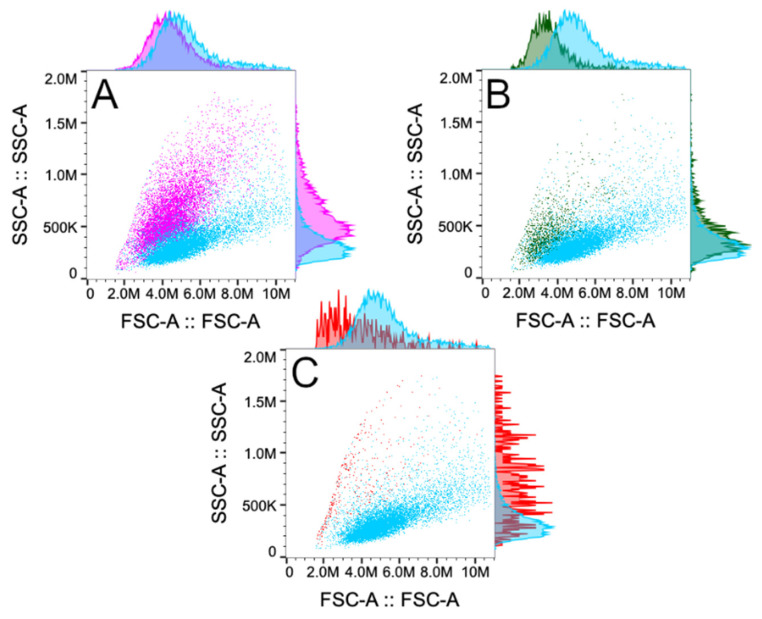
Used gating strategy of flow cytometry (forward scattering (FSC) vs. side scattering (SSC)) and the obtained cell population change in IRE (rose) compared to control (blue) (**A**), CaEP (green) compared to control (blue) (**B**), and BLM electrotransfer (red) compared to control (blue) (**C**).

After incubation of directly affected cells, the conditioned medium was collected and centrifuged twice. Then, 15,000 untreated cells were plated into a 24-well plate and conditioned medium (400 μL for each well) was poured onto unaffected cells and incubated at 37 °C for 48 h. After this, the growth medium was removed and 100 μL TrypLE Express Enzyme (Thermo Fisher Scientific, Carlsbad, CA, USA) was added to detach the adherent cells from plate surface. Then, the cells were collected into 1.5 Eppendorf tubes and measured with a flow cytometer (BD Accuri C6). Obtained results were analyzed with FlowJo software (10.8.2). Figure 7 shows the flow cytometry gating strategy (forward scattering (FSC) vs. side scattering (SSC)) and the obtained cell population change in IRE (rose), CaEP (green), and BLM electrotransfer (red) is compared to control (blue).

#### 4.4.4. Wound Healing Test

The wound healing or scratch test is employed to assess cell migration in vitro. This method is based on monitoring cell migration by creating a “wound” on a cell monolayer. Following exposure to electric fields, 90,000 cells were cultured in 200 μL growth medium for 48 h. Afterward, the growth medium was centrifuged twice, and the medium was transferred to unaffected cells. Cells that were not exposed to electric fields were seeded into a 24-well plate (100,000 cells per well) and cultured for 24 h in 200 μL growth medium. After 24 h of culturing, the medium was removed, and a line was scratched into the cell monolayer using a sterile pipette tip. Then, growth medium from the affected cells was applied on monolayer cells. Pictures were taken using an inverted light microscope (Kern OCO-255, Frankfurt, Germany) at 0, 24, and 48 h after transferring the affected medium. The wound healing size was quantified as a percentage using the ImageJ (2.14.0/1.54f) program with the Wound Healing Size Tool plugin [32].

### 4.5. Statistical Analysis

MS Excel (Redmond, WA, USA) were used for the statistical analysis. The figures show the average of at least three repetitions that were repeated in at least three individual experiments. Whiskers represent standard error of the mean (SEM). The *t*-test was used to assess whether there were statistically significant differences between experimental groups. The normality was evaluated by the Shapiro–Wilk test. When the *p*-values were less than 0.05, 0.01, or 0.001, the significance was indicated by *, **, or ***, respectively.

## 5. Conclusions

Several key findings regarding the bystander effect are presented here. First, by using three viability methods (metabolic activity, amount of cells, and capability to form colonies) we demonstrate that the bystander effect occurs not only in CHO cells (as previously reported by our group) but also in 4T1 cells, indicating that this phenomenon is not limited to a single cell line.

Additionally, short incubation times following irreversible electroporation result in an increased number of colonies compared to the control group in a clonogenic assay. This was shown by our group with CHO cells previously [20]. Therefore, here we indicate that this phenomenon is seen in more than one cell line. We hypothesize that this amount of colony increase is due to the initiation of cell migration, as evidenced by the observation of a larger number of smaller colonies.

Furthermore, we propose a novel approach utilizing conditioned media obtained from cells directly subjected to calcium electroporation, irreversible electroporation, and bleomycin electrotransfer. Our findings suggest that combining these conditioned media sources may withhold the bystander effect, by decreasing the viability of indirectly treated cells the same as with only one of the preferred electroporation-based treatments. This suggests the bystander effect may occur even when multiple electroporation-based treatments are applied.

Our results provide novel evidence that the bystander effect influences not only individual cellular processes but also the intricate interplay between cell migration and proliferation. This finding was demonstrably visualized using a wound healing assay.

## Data Availability

Data are available from the corresponding author P.R. on request.
